# Presence of mucin droplets during endoscopic submucosal dissection at the submucosa and beyond

**DOI:** 10.1055/a-2934-8320

**Published:** 2026-08-27

**Authors:** Luis Wong, Francesco Pindozzi, Raquel Muñoz-González, Cecilia Perelló, Andreina Padron, Harold Benites-Goñi, Hugo Uchima

**Affiliations:** 1 Research staff 196637 Foundation Institute of Research in Health Sciences Germans Trias i PujolBadalona87Spain; 2 Malattie dell'Apparato Digerente 18561 University Hospital CareggiFlorenceItaly; 3 Endoscopy Unit 16514 Hospital Universitari Germans Trias i PujolBadalonaSpain; 4 Pathology Department 16514 Hospital Universitari Germans Trias i PujolBadalona87Spain; 5 Pathology Department 16711 Centro Médico TeknonBarcelonaSpain; 6 Gastroenterology 33225 Universidad San Ignacio de LoyolaLima DistrictLima RegionPeru; 7 Endoscopy Unit 16711 Centro Médico TeknonBarcelonaSpain; 8 196637 Foundation Institute of Research in Health Sciences Germans Trias i PujolBadalonaSpain; 9 468625 CIBEREHDMadridCommunity of MadridSpain

Mucinous adenocarcinoma is an uncommon histological subtype of colorectal cancer accounting for 10–20% of all CRC cases, histologically defined by extracellular mucin and constituting more than half of the tumor 1 . It is associated with a higher risk (OR 2.26, *p* < 0.001) of lymphatic involvement in early stages of CRC 2 . The accumulation of mucin can be visualized endoscopically with a “droplet-like” appearance. When these droplets are identified in the resection plane during endoscopic submucosal dissection (ESD), it is essential to rule out mucinous adenocarcinoma 3 . Mucin could also be found in non-invasive lesions. We present two cases in which mucin droplets were observed during ESD, with different distribution and histology.

**Fig. 1:**
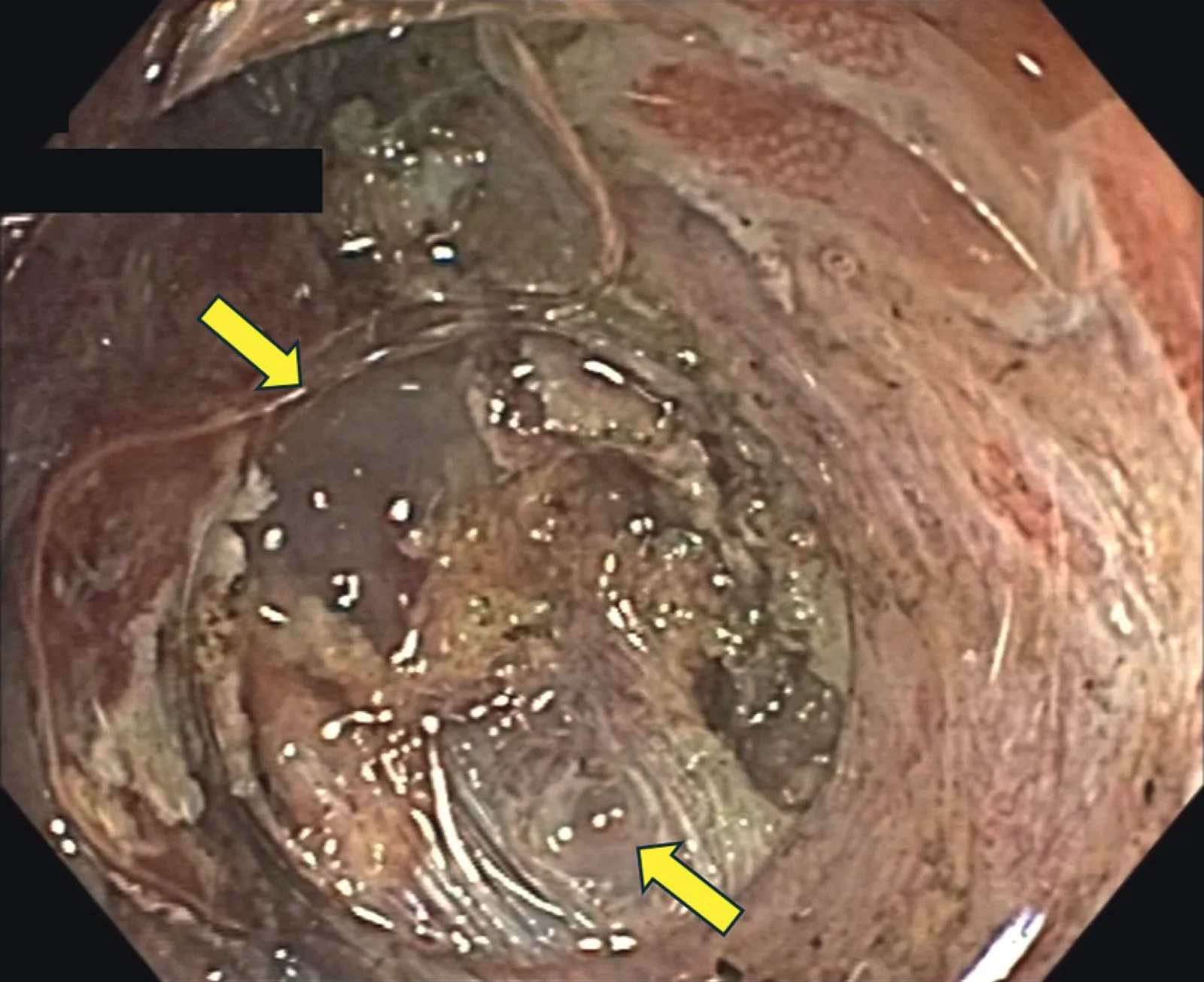
Mucin droplets in the submucosal plane of a 30-mm polyp with high-grade dysplasia.

**Fig. 2:**
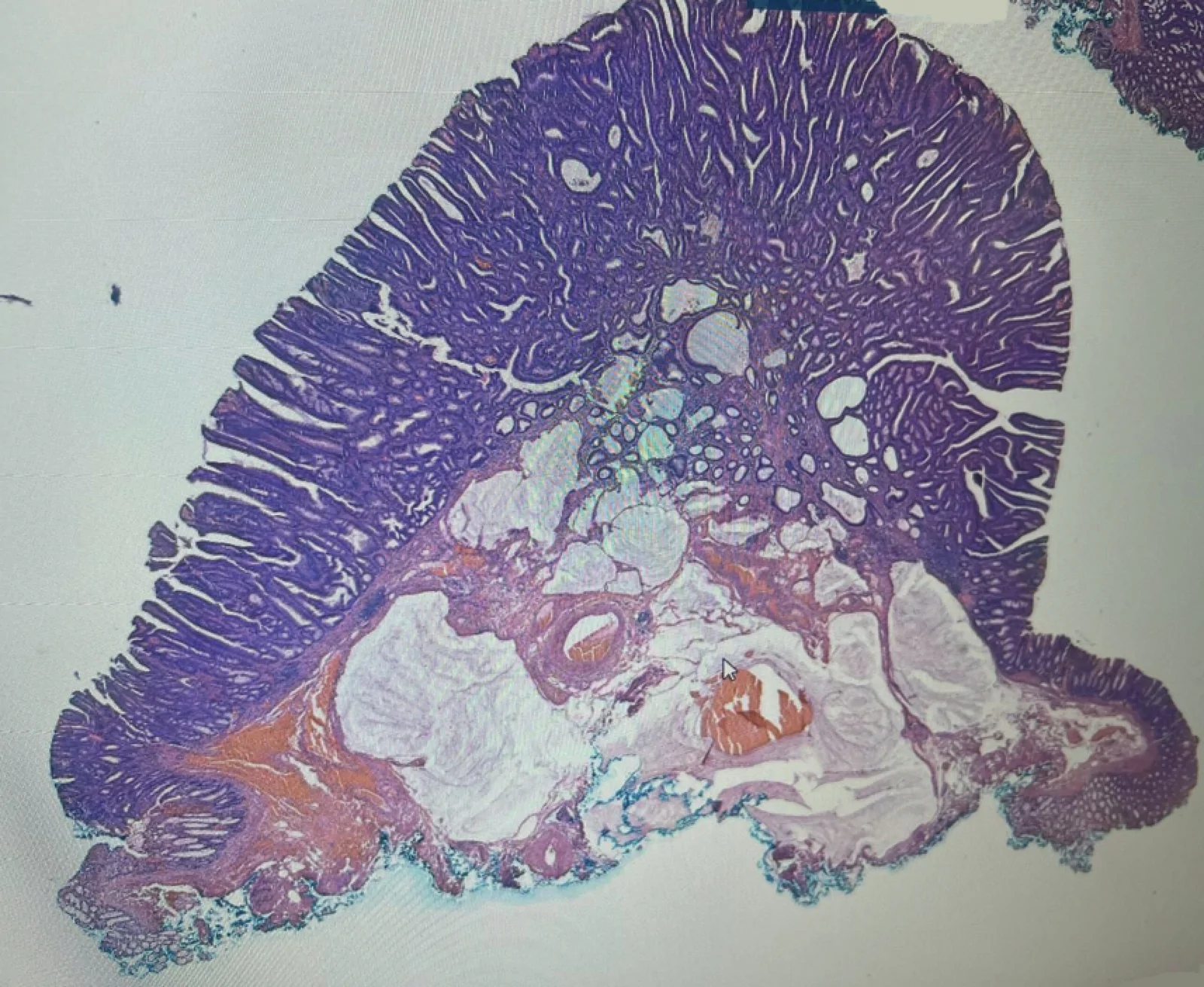
Pathological examination showed high-grade dysplasia with mucin-draining glands.

In the first case an 86-year-old woman with a 30-mm sessile lesion (Paris 0-Is, JNET 2b), at the hepatic flexure of the colon, was referred for ESD. During ESD, mucin droplets were observed in the submucosa ( Fig. 1 ), so the dissection plane was continued in the deep submucosal layer, below the mucin droplets, keeping the dissection as close as possible to the muscularis propria while carefully avoiding injury to the muscular layer. En bloc resection was achieved without procedural adverse events ( Video 1 ). Pathological examination showed high-grade dysplasia with mucin-draining glands ( Fig. 2 ). Because of the mucin droplets, a surveillance colonoscopy at 6 months was performed with biopsies showing no residual tissue.

**Video 1** Presence of mucin droplets during endoscopic submucosal dissection within the submucosal layer. Video URI: .


**Fig. 3:**
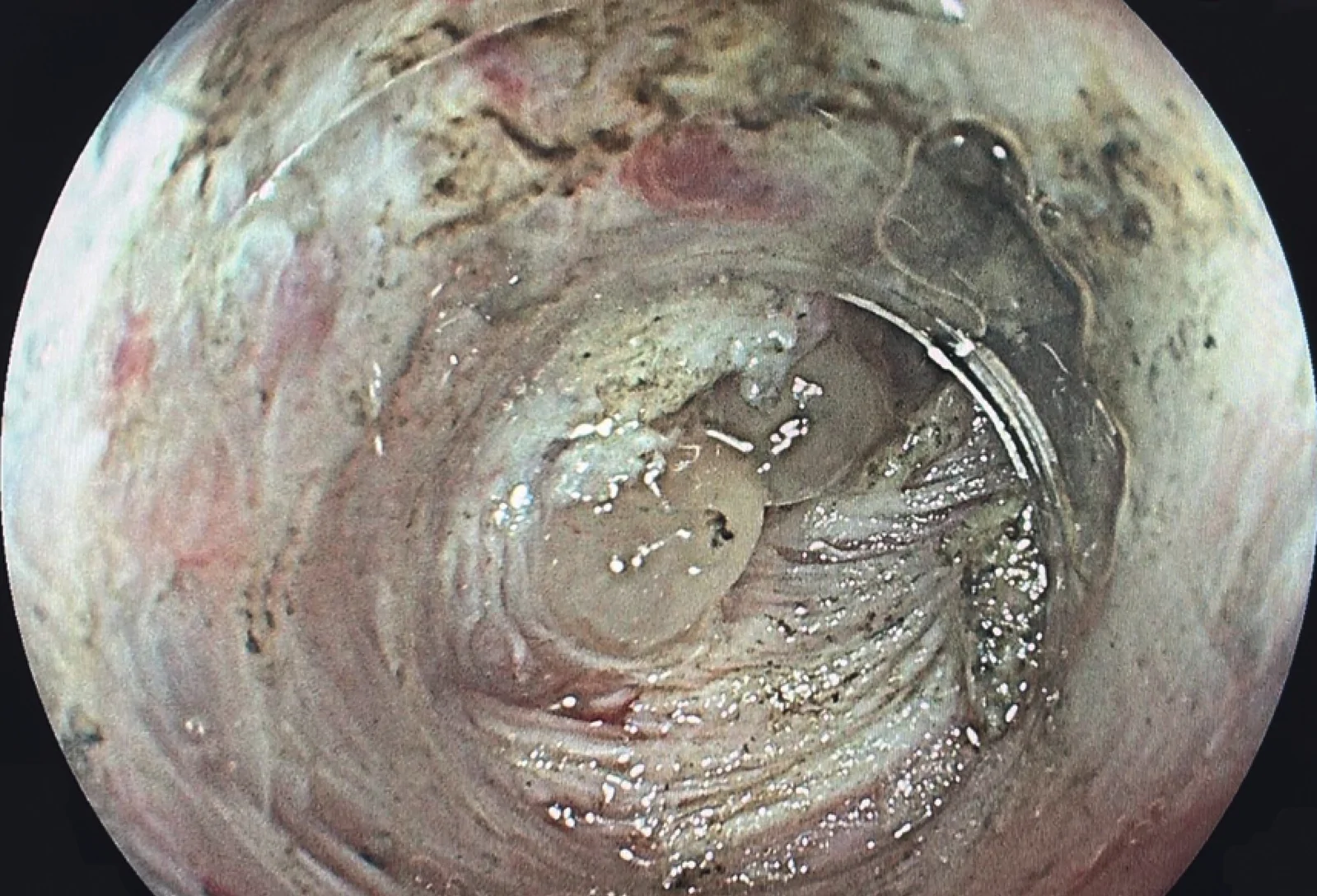
Mucin droplets in the submucosal plane and some between the fibers of the inner muscular layer of a mucinous adenocarcinoma.

In another case, a 57-year-old man was referred for ESD after recurrence of a piecemeal resection of a 30 mm sessile rectal lesion (Paris 0-Is, NICE 2, JNET 2a), without signs of deep submucosal invasion. During ESD, submucosal fibrosis was found with visualization of mucin droplets in the submucosa, between the fibers of the muscular layer ( Fig. 3 ), and in the deep muscularis propria at the scar after the en-bloc resection. Histology revealed a moderately differentiated mucinous adenocarcinoma infiltrating the submucosa, and surgical resection was recommended.

These two different cases demonstrate that the presence of mucin in the resection plane may be an endoscopic sign of mucinous adenocarcinoma, although it may also be present in non-invasive lesions. If the mucin seems to be confined to the submucosa, the endoscopic resection has curative potential.

Endoscopy_UCTN_Code_TTT_1AQ_2AD_3AD
